# Characterization of Biomarkers in Colorectal Cancer Liver Metastases as a Prognostic Tool

**DOI:** 10.3390/jpm11111059

**Published:** 2021-10-21

**Authors:** Sternschuss Michal, Goshen-Lago Tal, Perl Gali, Goldenfeld Miki, Brook Elana, Brenner Baroch, Kashtan Hanoch, Ben Aharon Irit, Haddad Riad

**Affiliations:** 1 Davidoff Cancer Center, Rabin Medical Center, Division of Oncology, Petah Tikva 69978, Israel; avrahami.michal@gmail.com (S.M.); GaliP@clalit.org.il (P.G.); brennerb@clalit.org.il (B.B.); 2 Division of Oncology, Rambam Health Care Campus, Haifa 3109601, Israel; T_goshenlago@rambam.health.gov.il (G.-L.T.); i_benaharon@rambam.health.gov.il (B.A.I.); 3 Sackler Faculty of Medicine, Tel Aviv University, Ramat Aviv, Tel Aviv 69978, Israel; goldenfeld@mail.tau.ac.il (G.M.); hkashtan@clalit.org.il (K.H.); 4 Rabin Medical Center, Department of Pathology, Petah Tikva 49100, Israel; elenabr1@clalit.org.il; 5 Rabin Medical Center, Division of Surgery, Beilinson Campus, Petah Tikva 49100, Israel; 6 Rappaport Faculty of Medicine, Technion, Haifa 3525428, Israel; 7 Carmel Medical Center, Department of Surgery B, Haifa 3436212, Israel

**Keywords:** colorectal cancer liver metastasis, hepatic surgery, prognosis, biomarkers, molecular signature

## Abstract

Background: Unfortunately, the majority of patients with colorectal cancer liver metastases (CRCLM) experience disease recurrence following hepatic surgery. The key challenge is therefore optimal patient selection, which currently relies on anatomical and clinical parameters. Exploring a potential molecular signature may be predictive for seeing a clinical benefit from CRCLM resection. Methods: Consecutive patients who underwent CRCLM resection at our medical center between 2006 and 2016 were divided into cohorts of “good prognosis” (GP) or “poor prognosis” (PP) based on the time interval between their resection and disease recurrence. Proteomic analysis was performed on the surgical specimen and correlation analysis was carried out with demographics and clinical outcomes. Results: Proteomic analysis revealed 99 differentially expressed proteins of which a third were associated with extracellular matrix (ECM) pathways as the matrix metalloproteinases (MMPs). Multivariate analysis yielded a statistically differential proteomic pattern between the cohort regardless of perioperative treatment. Conclusion: Our results indicate a different proteomic landscape in the cohort of patients who had a clinical benefit from CRCLM resection which appears to be correlated with ECM pathways. Further prospective studies are needed to define the role of ECM pathways in prognostics and patient selection for surgical procedures for CRCLM.

## 1. Introduction

Despite effective screening programs and comprehensive treatment, the mortality rate of colorectal cancer (CRC) remains high [1,2]. Approximately 25% of CRC patients are diagnosed with colorectal cancer liver metastases (CRCLM) and are defined as having synchronous CRCLM. Such synchronicity has been correlated with a poor prognosis [3].

Local therapies for CRCLM include surgical resection, stereotactic ablative body radiotherapy (SBRT), thermal ablation, selective internal radiotherapy (SIRT), bland or chemoembolization and hepatic arterial infusion [4]. For patients with limited disease, surgical resection remains the treatment of choice. Yet, the 5-year survival rates of patients treated surgically only reach to 30–50% and most patients experience a recurrence of the disease. The management of patients with CRCLM therefore remains challenging. The primary tumor location has been correlated with the prognosis after CRCLM resection. Namely, left-sided primary tumors have a significantly improved median overall survival though no superior recurrence free survival [5,6]. Another potential biomarker concerns the histopathologic growth patterns (HGP) of CRCLM; desmoplastic HGP are associated with improved survival compared with pushing and replacement HGP [7,8,9]. Nevertheless, although such clinical and pathological factors have been shown to be correlated with survival, patient selection criteria remain unclear. Since the majority of patients will experience recurrence, there is a need for superior surrogate prognostic and predictive biomarkers [10].

The introduction of predictive biomarkers such as KRAS, NRAS, BRAF and Microsatellite Instability (MSI) has provided the impetus to revisit the clinical approach for systemic treatment in metastatic CRC. Recently biomarkers, such as SMAD4 loss which is associated with a poor prognosis and a loss of immune infiltrate with an inverse response to treatment, have been identified yet not implemented into practical clinical considerations. Nevertheless, none have been shown to correlate with a better response to liver-directed therapies for CRCLM [11].

The recurrence of liver metastases following hepatic resections may derive from persistent residual microscopic disease. We therefore hypothesized that the interaction of hepatic stroma with macro and micro metastases and the activation of the immune system may play a key role in the eradication of residual disease (synergistic to systemic treatment). This may subsequently be translated into long-term remission following hepatectomy.

In this study we aimed to explore the molecular landscape of CRCLM and their adjacent hepatic stroma. This was in order to define a specific characteristic signature for patients who remain free of recurrence for more than 12 months after surgical resection of CRCLM compared to patients who experience early recurrence (within 12 months) after surgery. Accordingly, we aimed to evaluate potential biomarkers of the molecular signature that may be predictive for effective patient selection.

## 2. Materials and Methods

### 2.1. Patients

Consecutive patients with CRCLM who were treated at the department of surgery at Rabin Medical Center (RMC) between 2006 and 2016, and for whom formalin fixed frozen paraffin embedded (FFPE) surgical samples of liver metastases were available, were included in the study. Patients with extrahepatic metastases or insufficient clinical data were excluded. The remaining patients were dichotomized into “good prognosis” (GP) and “poor prognosis” (PP) cohorts based on their clinical and radiological evaluation of the interval to recurrence. The cutoff point of 12 months was chosen based on previous reports that found a correlation between the timing of recurrence and survival [12]. Budget constraints allowed the inclusion of 58 patients in this study; 29 patients in each cohort, and therefore required patient selection. In order to maximize the differentiation between the two cohorts, patients in the PP cohort were further dichotomized into very early recurrence (<6 months) and early recurrence (6–12 months) groups. In the final analysis we included all patients with a very early recurrence in addition to randomly selected patients with early recurrence in the PP cohort and randomly selected patients in the GP cohort. Clinical data was retrieved from the RMC’s Davidoff Cancer Center registry regarding patient demographics, histological traits of the tumor, cancer routine markers, chemotherapy and biological treatments, tumor location, disease recurrence, and survival. This study was approved by the medical center’s ethics committee (0456-16RMC) and all procedures were performed in accordance with the institution’s policy.

### 2.2. Proteomic Analysis

Proteomic analysis was performed at the Smoler Proteomics Center, Technion Institute, Israel. Five slides from each FFPE sample were micro-dissected to isolate and collect only the tumor cells of interest for analysis. The micro-dissected FFPE tumor tissue was then subjected to Liquid Tissue^®^ processing to solubilize the tumor tissues. The patented Liquid Tissue^®^ technology ensures that formalin cross-links are reversed and all the proteins in the tumor tissue are solubilized. Tissues were homogenized with Omni-Th homogenizer in Urea buffer containing: 8 M Urea, 400 mM Ammonium bicarbonate, and 10 mM DTT. Homogenates were sonicated (5′, [10/10 on/off pulses], 90% energy. Sonics Vibra-Cell (Sonics & Materials, Inc., Newtown, CT, USA) and briefly centrifuged to pellet insoluble debris. The protein amount was estimated using Bradford readings. Then, 20 ug protein from each sample were reduced with DTT (60 °C for 30 min), modified with 40 mM iodoacetamide in 100 mM ammonium bicarbonate (in the dark, RT) and digested in 2 M Urea, 100 mM ammonium bicarbonate with modified trypsin (Promega), overnight at 37 °C with a 1:50 enzyme-to-substrate ratio. An additional second digestion with trypsin was done for 4 h at 37 °C with a 1:100 enzyme-to-substrate ratio. The tryptic peptides were desalted using C18 tips (Harvard) dried and re-suspended in 0.1% formic acid. The peptides were resolved by reverse-phase chromatography on 0.075 × 300-mm fused silica capillaries (J&W) packed with Reprosil reversed phase material (Dr Maisch GmbH, Germany). The peptides were eluted with linear 180 min’s gradient of 5 to 28%, 15 minutes’ gradient of 28 to 95%, and 25 min at 95% acetonitrile with 0.1% formic acid in water at flow rates of 0.15 μL/min. Mass spectrometry (MS) was performed by Q Exactive plus mass spectrometer (Thermo) in a positive mode using a repetitively full MS scan followed by High Collision Dissociation (HCD) of the 10 most dominant ions selected from the first MS scan. The mass spectrometry data was analyzed using the MaxQuant software 1.5.2.4. (www.maxquant.org) for peak picking identification and quantitation using the Andromeda search engine, searching against the human UniProt database with mass tolerance of 20 ppm for the precursor masses and 20 ppm for the fragment ions. Oxidation on methionine proline and lysine, and protein N-terminus acetylation were accepted as variable modifications and carbamidomethyl on cysteine was accepted as static modifications. Minimal peptide length was set to six amino acids and a maximum of two miscleavages was allowed. Peptide- and protein-level false discovery rates (FDRs) were filtered to 1% using the target-decoy strategy. Protein tables were filtered to eliminate identifications from the reverse database, and common contaminants and single peptide identifications. The data was quantified by label free analysis, using the same software based on extracted ion currents (XICs) of peptides, enabling the quantitation from each liquid chromotography/MS run for each peptide identified in any of experiments. The mass spectrometry proteomics data were sent to the ProteomeXchange Consortium and deposited in the PRIDE [13] partner repository with the dataset identifier PXD022613.

### 2.3. Statistical Analysis

For the proteomic analysis, the normal test was used on the fold change (using a log scale) to compare the expression of each protein between the GP and the PP groups. The *p*-values across all proteins were adjusted to control the FDR. The 0.05 level was used for significance. Multivariate logistic model was used to test the clinical effects on the probability for a one-year recurrence. Multivariate Cox proportional hazard regression was used to test the clinical effects on one-year recurrence free survival. Cox regression was also used to test the effect of each protein on this survival, adjusting the *p*-values across all proteins to control the FDR.

## 3. Results

Electronic medical records identified 259 patients with CRCLM who were treated at our medical center’s surgery department during the study period, and for whom formalin fixed frozen paraffin embedded (FFPE) surgical samples of liver metastases were available, were identified through electronic medical records. Of these, 136 patients were excluded due to insufficient clinical data or extrahepatic metastases prior to liver resection. The remaining 123 patients were dichotomized into GP (N = 68) and PP (N = 55) cohorts. The median time to recurrence was 5.6 months in the PP cohort and 23.6 months in the GP cohort.

In the final analysis, we included 29 randomly selected patients in the GP cohort, while the PP cohort consisted of all 19 patients with a very early recurrence and another 10 randomly selected patients with early recurrence. See Supplementary Materials Figure S1 for the patient inclusion flow-chart.

### 3.1. Clinical Characteristics

Patient characteristics are described in Table 1. The PP cohort had a male predominance of 62% in contrast to 48% in the GP cohort, although this was not statistically significant. Family history of malignancy was found to be a factor for a poor prognosis from CRCLM. Such family history was found in 36% of patients in the GP compared with 56% in the PP, where the hazard ratio for recurrence within 1 year from surgery in patients without family history was 0.3 [CI:(0.11,0.78), *p* = 0.0144] (Supplementary Materials Figure S2).

The primary tumor location did not significantly differ between the two groups: 34% and 31% right-sided tumors in the GP and PP respectively. Although synchronicity is considered a poor prognostic factor, 76% of patients in the GP group had synchronous disease as compared with 52% in the PP, though this was not statistically significant.

The PP cohort had the same median number of hepatic metastases (1) as the GP, with a higher percentage of bi-lobar disease (28% compared to 14%), and slightly larger metastases (median 29 mm for the largest lesion compared with 23.5 mm). These differences were not statistically significant. Supplementary Table S1 shows all the assessed prognostic factors.

The vast majority of our cohort received systemic chemotherapy (only 10% of the GP and 17% of the PP did not receive chemotherapy). Nevertheless, in the PP cohort only 35% received perioperative chemotherapy compared with 55% in the GP cohort, though this did not reach statistical significance. The different treatment schedules of the two cohorts are described in Table 1. Interestingly, avoidance of neoadjuvant chemotherapy (either as part of a perioperative therapy or as neoadjuvant only therapy) was found to be significant in a multivariate analysis with a hazard ratio for recurrence within 1 year from surgery of 3.83 [CI:(1.12,13.06), *p*-value = 0.032] (Supplementary Materials Figure S3).

The choice of treatment protocol was different between the cohorts with the majority of GP treated with oxaliplatin-based regimens (88%) and bevacizumab (81%), whereas in the PP only 58% received oxaliplatin-based chemotherapy (the rest were treated with irinotecan-based chemotherapy or 5FU alone) and were less likely to receive bevacizumab (58%). These differences were not statistically significant.

### 3.2. Proteomics

Over 3700 proteins were identified using proteomic analysis. Expression levels were compared between the two cohorts. In 99 proteins, expression levels were significantly different between the two groups (Supplementary Materials Table S2). These proteins were further grouped to common pathways using the ‘Gorilla’ software. The protein expression is described in Table 2. Differences in proteomic profile appeared statistically significant in a multivariate analysis regardless of chemotherapy administration.

#### 3.2.1. ECM Pathway

Thirty-four proteins which play a role in several pathways of the extracellular matrix reached statistical significance. A key player was the matrix metalloproteinases (MMPs). Several MMPs were upregulated in the PP: MMP7 by 4.37% (*p* = 0.006) and Dehydropeptidase 1 (DPEP1) by 4.25% (*p* = 0.009). Other MMPs were downregulated: MMP12 by 4.53% (*p* = 0.0003), Meprin-a (MEP1A) by 6.89%, (*p* < 0.001), aminopeptidase A (APA or ENPEP) by 3.35% (*p* = 0.021), and A disintegrin and metalloproteinase decysin 1 (ADAMDEC 1) by 3.87% (*p* = 0.004). Other proteins involved in ECM related pathways that were downregulated in the PP compared with the GP include lysyl oxidase like 1 (LOXL1) by 4.26% (*p* < 0.001) and defensin alpha 5 paneth cell-specific (DEFA5) by 5.5% (*p* < 0.001), while insulin-like growth factor binding protein 2 (IGFBP2) was upregulated by 5.56% (*p* < 0.001).

#### 3.2.2. DNA Replication and Repair Pathways

MSH2, a member of the mismatch repair (MMR) complex, was upregulated by 5.5% (*p* < 0.001) in the PP cohort compared with the GP; Minichromosome maintenance 4 (MCM4) was also upregulated by 5.16% (*p* < 0.001). We could not detect any major differences in DNA repair pathways between the two groups.

#### 3.2.3. Immune Pathway

Several components of the immune pathway were downregulated in the PP cohort compared with the GP, possibly reflecting the effect of the immune response in the two cohorts. The statistically significant downregulated proteins included Complement component 5 (C5) by 4.2% (*p* = 0.001); Complement C1r subcomponent-like protein (C1RL) by 3.05% (*p* = 0.047); Complement component C8 alpha chain (C8A) by 5.4% (*p* < 0.001); Soluble CD163 by 4.3% (*p* < 0.001); chymase 1 expressed in mast cells by 3.3% (*p* = 0.02), and major histocompatibility complex (HLA-B) by 5.6% (*p* < 0.001).

Interestingly, Homebox protein CDX2, which has been associated with CRC aggressiveness, was not found to be significantly different between the two groups.

## 4. Discussion

We hypothesized that tumor unique characteristics and potential crosstalk with adjacent hepatic stroma may account for effective residual tumor eradication post resection of CRCLM regardless of perioperative systemic treatment. Comparing the baseline characteristics of the two cohorts (GP vs. PP) revealed no significant differences in demographics (sex and age). Furthermore, no correlation was found for habits (smoking and alcohol) and tumor characteristics (primary tumor location, KRAS status, number of liver metastases at diagnosis). Liver metastases synchronicity was also not associated with poorer prognosis. Patient characteristics were similar between the two cohorts implying the two groups were demographically balanced. Nevertheless, a lack of family history of malignancy was associated with a higher recurrence within one year compared with patients with a positive family history.

The impact of systemic treatment was analyzed and revealed differences between the two cohorts. Whereas perioperative treatment was indeed more prevalent in the GP cohort, in a multivariate analysis the differential gene expression pathway was significant regardless of perioperative treatment. This observation correlates to former evidence indicating that the addition of chemotherapy to surgical resection improves clinical outcome, however the optimal schedule in upfront resectable patients remains uncertain [14,15,16]. A lack of neoadjuvant treatment was also associated with a worse outcome. This observation may represent the clinical benefit of early treatment of micro-metastatic disease. The choice of chemotherapy regimen and biological agents differed between the cohorts with higher rates of irinotecan-based therapy in the PP and higher rates of bevacizumab in the GP, though no statistically significant conclusion could be drawn.

Evaluating the proteomic landscape of the two cohorts revealed 99 proteins that were differentially expressed, a third of which are associated with the ECM. However, we could not delineate a clear correlation between the ECM signature and clinical traits of the cohorts due to the limited sample size. We discuss below the current evidence regarding these proteins’ role in invasiveness and metastases in CRC and other cancer types.

### 4.1. Matrix Metalloproteinase

**Figure 1:**
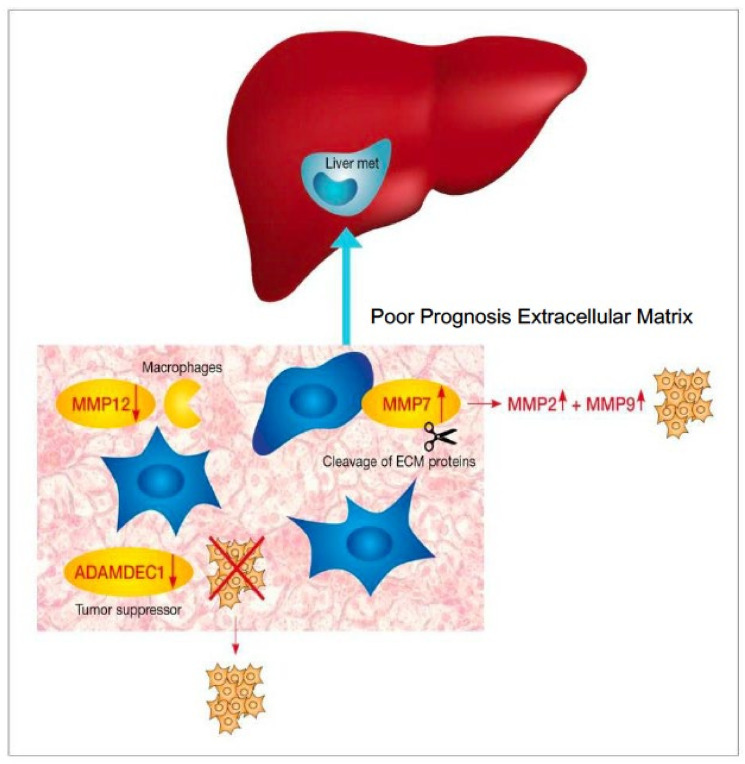
Scheme showing the “poor prognosis” microenvironment significant signature. In the “poor prognosis” microenvironment there is (i) Upregulation of MMP7 which promotes cancer invasion by proteolytic cleavage of ECM proteins and activation of cell proliferation through upregulation of MMP2 and MMP9 and (ii) Downregulation of the protective effect of MMP12 and the tumor suppressor, ADAMDEC1.

Currently published data describes a correlation between several proteins associated with the ECM and CRC aggressiveness. MMPs, a large family of zinc dependent endopeptidases, are known to play a crucial role in the degradation and remodeling of ECM and the processing of other bioactive molecules. As such, they were shown to contribute to tumor invasiveness and metastases in several cancer types including CRC, while changes in the expression of several members of this family of proteins were shown to be associated with increased mortality risk [17,18,19,20]. Despite encouraging results, the use of these biomarkers remains investigational. We did find a differential MMP signature between PP and GP. The proposed pathway displayed in PP is presented in Figure 1. Matrilysin, also known as MMP7, promotes cancer invasion in several processes including proteolytic cleavage of ECM proteins. While activation of other MMPs, including MMP2 and MMP9 overexpression and high levels of serum MMP7 levels, have been linked to CRC progression and decreased survival in advanced CRC [18,20].

Our findings indicating higher levels of MMP7 in the PP group support previously published data, suggesting that MMP7 indeed plays a role in advanced metastatic CRC progression. Accordingly, serum MMP7 may be an appealing secreted biomarker that should be further studied in a prospective setting. Another member of the MMP family is MMP12, also known as Metalloelastase, which is a multi-substrate degrader predominantly expressed in macrophages. MMP12 has been shown to have a protective effect in CRC, where higher expression levels have been associated with primary tumors without hepatic metastases and better survival compared to CRCLM [21]. MMP12’s effect on tumor progression may be mediated via its effect on the tumor vasculature. MMP12 is reciprocally expressed with vascular endothelial growth factor (VEGF) and is correlated with increased levels of angiostatin, an endogenous angiogenesis inhibitor [17,21]. Interestingly, Klupp et al. [22] found that higher serum levels of MMP12 were associated with a worse prognosis. The reason for these different observations isn’t clear. Our results indicate decreased levels of MMP12 in the PP group suggesting the protective effect extends to the metastatic phase as well.

The ADAM family of proteins are involved in various biological events such as cell adhesion, cell fusion, cell migration, membrane protein shedding and proteolysis [23,24]. ADAMDEC1 is a known tumor suppressor gene that has been previously studied in CRC. ADAMDEC1 has been shown to be inversely expressed with the degree of disease aggressiveness, with a more prominent effect in patients without family history of CRC.

A potential aspect for further research is the correlation with HGP, as HGP subgroups are associated with a different ECM profile (e.g., collagen-rich stroma in desmoplastic growth patterns). Therefore, might relay on different proteolysis and spreading mechanisms [25], as well as different immune responses.

### 4.2. Other ECM Pathways

LOXL1 is a member of the LOX family of proteins, a copper-dependent amine oxidase that catalyzes the crosslinking of collagens and elastin in the ECM as well as being involved in intracellular and nuclear processes. Higher levels of LOX, and specifically nuclear expression, have been associated with metastases and poor prognosis [26]. In our study, LOXL1 was found to be downregulated in the PP cohort, contradicting previously published data. A possible explanation for this discrepancy may derive from our referral to total protein levels and not specifically nuclear location.

DNA replication and repair pathways MSH2 is a member of the mismatch repair proteins. Most published data regards the loss of its expression in the context of microsatellite instable (MSI) tumors and Lynch syndrome. However, data is scarce regarding isolated MSH2 expression or overexpression. Interestingly, we found overexpression of MSH2 in the PP group compared with the GP. It is not clear whether there is a correlation between this finding and the difference in family history between the cohorts. MCM4 belongs to the MCM protein complex which plays a role in the initiation of DNA replication and DNA unwinding [27]. Members of MCM complex are present in proliferating cells and overexpression has been described in several cancer types. A recently published CRC study described MCM4 as part of a novel four gene prognostic model [28]. Our results indicating overexpression in the PP cohort further supports the role of MCM4 as a marker for poor prognosis.

### 4.3. Immune Pathways

Understanding the role of the host immune system in tumor progression is an evolving research theme. Mounting evidence has correlated tumor infiltrating immune cells and the inflammatory response with clinical outcome [6]. In this study we found several significant differences in factors associated with the immune response. Complement 5 (C5) is a potent pro-inflammatory immune mediator that plays a role in innate immunity. Previous studies in cell lines found that c5 modulates tumor inflammation in CRC and has a pro-metastatic effect, while inhibition of the c5a receptor signaling severely impairs tumor metastasis [29]. Our results contradict these observations since we found decreased levels of C5 in the PP group.

Soluble CD163 is a monocyte-macrophage scavenger receptor and is regarded as a marker of macrophage activation. High serum levels of sCD163 have been described in several malignancies and have been shown to correlate with worse overall survival in gastric cancer patients [30]. Conversely, our cohort shows down regulation of sCD163 in the PP cohort, possibly reflecting lower levels of macrophage activation.

Mast cells are tissue-resident immune cells that modulate the immune response. Their role in cancer progression is complex. There is evidence that they promote angiogenesis, proteolytic activity affecting ECM proteins, and cancer cell invasion and migration. Whereas other studies have suggested an antitumoral effect of mast cells. In our study, we found down regulation of chymase1 in the PP cohort, possibly indicating a pro-tumoral effect. Further research is warranted to define the role of the immune system in the tumor microenvironment in the context of hepatic metastases.

Our study has several limitations. The small sample size and the fact that we didn’t perform corresponding transcriptome and genome analysis. Nevertheless, our results indicate a different proteomic landscape in the cohort of patients who had a clinical benefit from CRCLM resection which appears to be correlated with ECM pathway.

## 5. Conclusions

In the era of precision medicine, the clinical approach to CRCLM still relies on “traditional” factors including anatomical considerations and the number of metastases, there has been little consideration for molecular biomarkers. We found in this retrospective study that a unique signature of ECM proteins may serve as a ground for further large prospective research in order to validate the value of this proteomic signature and its role as a prognostic marker for CRCLM resection. Identifying potential biomarkers in the adjacent hepatic stroma in future prospective studies may allow for better selection of suitable candidates for CRCLM resection.

## Figures and Tables

**Table 1:** Patients’ Characteristics.

			GP	PP	*p*-Value
Median follow up (range) months			64 (33–149)	34 (11–153)	NS
Dem	Gender	Male Female	14 (48%) 15 (52%)	18 (62%) 11 (38%)	NS
	Median age at metastatic disease dg. (Range) years		62 (45–81)	64 (34–85)	NS
	Family history of malignancy		10 (36%)	16 (56%)	0.0144
Primary tumor	Side of primary tumor	Right-sided tumors Left-sided tumors	10 (34%) 19 (66%)	9 (31%) 20 (69%)	NS
	Primary tumor differentiation	Well Moderate Poor Unknown	4 (14%) 23 (79%) 1 (3.5%) 1 (3.5%)	6 (21%) 21 (72%) 1 (3.5%) 1 (3.5%)	NS
Liver mets	Median number of liver mets at dg. (Range)		1 (1–4)	1 (1–6)	NS
	Median size of largest met. (Range) mm		23.5 (10–120)	29 (8–160)	NS
	Bilobar liver mets. Unilobar liver mets		4 (14%) 25 (86%)	8 (28%) 21 (72%)	NS
Metastases appearance	Metachronous disease		7 (24%)	14 (48%)	NS
	Stage at diagnosis: I II III Unknown		4/7 (57%) 3/7 (43%)	1/14 (7%) 8/14 (57%) 4/14 (29%) 1/14 (7%)	NS
	Adjuvant therapy for localized disease Yes No		6/7 (86%) 1/7 (14%)	10/14 (71%) 4/14 (29%)	NS
	Synchronous disease		22 (76%)	15 (52%)	NS
	Surgery for primary tumor: Prior to CRCLM resection After CRCLM resection Simultaneous procedure		11/22 (50%) 2/22 (9%) 9/22 (41%)	10/15 (67%) 2/15 (13%) 3/15 (20%)	NS
Systemic treatment	Yes No		26 (90%) 3 (10%)	24 (83%) 5 (17%)	NS
	Neoadjuvant (either alone or perioperative) Adjuvant (either alone or perioperative) Perioperative		21/26 (81%) 21/26 (81%) 16/26 (62%)	23/24 (96%) 11/24 (46%) 10/24 (42%)	0.032 NS NS
	Type	Chemotherapy- Oxaliplatin based Irinotecan based 5FU only Biological agent- Bevacizumab Cetuximab none	23/26 (88%) 3/26 (12%) 0 21/26 (81%) 2/26 (8%) 3/26 (11%)	14/24 (58%) 9/24 (38%) 1/24 (4%) 14/24 (58%) 0 10/24 (42%)	NS NS
	Completed six months of therapy	Yes No Unknown	21/26 (81%) 4/26 (15%) 1/26 (4%)	19/24 (79%) 3/24 (13%) 2/24 (8%)	NS
Outcome	Median time to recurrence (months)		23.6	5.6	
	Subsequent treatment	Surgery: Yes No unknown Radiotherapy: Yes No unknown Systemic therapy- Yes No Unknown	14 (48%) 12 (41.5%) 3 (10.5%) 5 (17%) 22 (76%) 2 (7%) 18 (62%) 9 (31%) 2 (7%)	12 (41.5%) 16 (55%) 1 (3.5%) 6 (21%) 21 (72%) 2 (7%) 24 (83%) 2 (7%) 3 (10%)	NS

No.—Number, Pt.—Patient, Mets—Metastases, Dg—Diagnosis, NS—not significant, Dem—Demographics. GP—good prognosis, PP—poor prognosis.

**Table 2:** Significant proteins according to proteomic analysis and their related pathways of expression—A normal test was used on the fold change (presented on a log scale) to compare the expression of each protein between the “good prognosis” and the “poor prognosis” groups. *p*-values across all proteins were adjusted to control the false discovery rate (FDR).

Gene Name	Protein Name	Ratio of Protein Expression in Tumor Samples of “Bad Prognosis” vs. “Good Prognosis”	Fold Change (Bad Prognosis vs. Good Prognosis)	FDR Adjusted *p*-Value	Extracellular Space	Metallo Peptidase Activity	DNA Replication and Repair	Immune System
AKR1B10	Aldo-keto reductase family 1 member B10	Down	0.95	1.2 × 10^−4^	V			
APOB	apolipoprotein b	Down	0.97	2.0 × 10^−2^	V			
C1RL	Complement C1r subcomponent-like protein	Down	0.97	4.7 × 10^−2^	V			V
C5	complement component 5	Down	0.96	1.1 × 10^−3^	V			V
C8A	Complement component C8 alpha chain	Down	0.95	3.2 × 10^−6^	V			V
CD163	Soluble CD163	Down	0.96	8.5 × 10^−4^	V			V
CHGA	Chromogranin-A	Up	1.05	5.6 × 10^−4^	V			
CMA1	chymase 1, mast cell	Down	0.97	2.4 × 10^−2^	V			V
DEFA5	Defensin-5	Down	0.94	1.9 × 10^−6^	V			
GCG	Glucagon	Down	0.93	4.7 × 10^−9^	V			
GP2	Pancreatic secretory granule membrane major glycoprotein GP2	Down	0.96	7.2 × 10^−3^	V			
HLA-B	HLA class I histocompatibility antigen, B-40 alpha chain	Down	0.94	1.3 × 10^−6^	V			V
HSD17B13	17-beta-hydroxysteroid dehydrogenase 13	Down	0.96	4.1 × 10^−3^	V			
IGFBP2	Insulin-like growth factor-binding protein 2	Up	1.06	7.5 × 10^−5^	V			
IGLV3–10	immunoglobulin lambda variable 3–10	Up	1.05	2.2 × 10^−4^	V			
KRT31	keratin 31	Down	0.96	9.9 × 10^−3^	V			
KRT85	keratin 85	Down	0.95	1.6 × 10^−4^	V			
LEFTY1	Left-right determination factor 1	Up	1.07	1.7 × 10^−7^	V			
LFNG	Beta-1,3-N- acetylglucosaminyltransferase lunatic fringe	Down	0.93	5.2 × 10^−10^	V			
LOXL1	Lysyl oxidase homolog 1	Down	0.96	8.5 × 10^−4^	V			
MXRA5	Matrix-remodeling-associated protein 5	Down	0.96	7.4 × 10^−3^	V			
OLFM4	Olfactomedin-4	Down	0.93	4.5 × 10^−11^	V			
OLFML1	Olfactomedin-like protein 1	Up	1.05	2.5 × 10^−4^	V			
OSCAR	Osteoclast-associated immunoglobulin-like receptor	Down	0.97	3.8 × 10^−2^	V			
PROM1	Prominin-1	Down	0.94	1.7 × 10^−6^	V			
PXDN	Peroxidasin homolog	Down	0.95	1.8 × 10^−4^	V			
TNFSF13	Tumor necrosis factor ligand superfamily member 13	Down	0.97	3.0 × 10^−2^	V			
DPEP1	Dipeptidase 1	Up	1.04	9.1 × 10^−3^	V	V		
MEP1A	Metalloendopeptidase; Meprin A subunit alpha	Down	0.93	5.2 × 10^−10^	V	V		
MMP12	Macrophage metalloelastase	Down	0.95	2.7 × 10^−4^	V	V		
MMP7	matrix metallopeptidase 7	Up	1.04	6.4 × 10^−3^	V	V		
ADAMDEC1	adam-like, decysin 1	Down	0.96	4.1 × 10^−3^	V			
ENPEP	Glutamyl aminopeptidase	Down	0.97	2.1 × 10^−2^	V			
METAP1	Methionine aminopeptidase 1	Down	0.96	3.2 × 10^−4^	V			
MSH2	MutS homolog 2	Up	1.05	1.0 × 10^−4^			V	
MCM4	Minichromosome maintenance 4	Up	1.05	3.4 × 10^−4^			V	

## Data Availability

The mass spectrometry proteomics data have been deposited to the ProteomeXchange Consortium via the PRIDE partner repository and is available with the dataset identifier PXD022613.

## Supplementary Materials

The following are available online at https://www.mdpi.com/article/10.3390/jpm11111059/s1, Figure S1: Patients inclusion flow-chart, Figure S2: KM Survival Curve according to patient’s family history, Figure S3: KM Survival Curve according neoadjuvant treatment Table S1: List of assessed prognostic factors, Table S2: List of significantly expressed proteins.Please check if the individual contribution of each co-author has been stated.

## References

[1] Ferlay J., Soerjomataram I., Dikshit R., Eser S., Mathers C., Rebelo M., Parkin D.M., Forman D., Bray F. (2015). Cancer incidence and mortality worldwide: Sources, methods and major patterns in GLOBOCAN 2012. Int. J. Cancer.

[2] Siegel R., Naishadham D., Jemal A. (2013). Cancer statistics, 2013. CA Cancer J. Clin..

[3] Adam R., de Gramont A., Figueras J., Kokudo N., Kunstlinger F., Loyer E., Poston G., Rougier P., Rubbia-Brandt L., Sobrero A. (2015). Managing synchronous liver metastases from colorectal cancer: A multidisciplinary international consensus. Cancer Treat. Rev..

[4] Van Cutsem E., Cervantes A., Adam R., Sobrero A., van Krieken J.H., Aderka D., Aguilar E.A., Bardelli A., Benson A., Bodoky G. (2016). ESMO consensus guidelines for the management of patients with metastatic colorectal cancer. Ann. Oncol..

[5] Creasy J.M., Sadot E., Koerkamp B.G., Chou J.F., Gonen M., Kemeny N.E., Saltz L.B., Balachandran V.P., Peter Kingham T., DeMatteo R.P. (2018). The Impact of Primary Tumor Location on Long-Term Survival in Patients Undergoing Hepatic Resection for Metastatic Colon Cancer. Ann. Surg. Oncol..

[6] Donadon M., Lleo A., Di Tommaso L., Soldani C., Franceschini B., Roncalli M., Torzilli G. (2018). The shifting paradigm of prognostic factors of colorectal liver metastases: From tumor-centered to host immune-centered factors. Front. Oncol..

[7] Van Dam P.J., Van Der Stok E.P., Teuwen L.A., Van Den Eynden G.G., Illemann M., Frentzas S., Majeed A.W., Eefsen R.L., Coebergh Van Den Braak R.R.J., Lazaris A. (2017). International consensus guidelines for scoring the histopathological growth patterns of liver metastasis. Br. J. Cancer.

[8] Buisman F.E., van der Stok E.P., Galjart B., Vermeulen P.B., Balachandran V.P., van den Braak R.R.J.C., Creasy J.M., Höppener D.J., Jarnagin W.R., Kingham T.P. (2020). Histopathological growth patterns as biomarker for adjuvant systemic chemotherapy in patients with resected colorectal liver metastases. Clin. Exp. Metastasis.

[9] Han Y., Chai F., Wei J., Yue Y., Cheng J., Gu D., Zhang Y., Tong T., Sheng W., Hong N. (2020). Identification of Predominant Histopathological Growth Patterns of Colorectal Liver Metastasis by Multi-Habitat and Multi-Sequence Based Radiomics Analysis. Front. Oncol..

[10] Kanas G.P., Taylor A., Primrose J.N., Langeberg W.J., Kelsh M.A., Mowat F.S., Alexander D.D., Choti M.A., Poston G. (2012). Survival after liver resection in metastatic colorectal cancer: Review and meta-analysis of prognostic factors. Clin. Epidemiol..

[11] Wasserman I., Lee L.H., Ogino S., Marco M.R., Wu C., Chen X., Datta J., Sadot E., Szeglin B., Guillem J.G. (2019). Smad4 loss in colorectal cancer patients correlates with recurrence, loss of immune infiltrate, and chemoresistance. Clin. Cancer Res..

[12] Takahashi S., Konishi M., Nakagohri T., Gotohda N., Saito N., Kinoshita T. (2006). Short time to recurrence after hepatic resection correlates with poor prognosis in colorectal hepatic metastasis. Jpn. J. Clin. Oncol..

[13] Perez-Riverol Y., Csordas A., Bai J., Bernal-Llinares M., Hewapathirana S., Kundu D.J., Inuganti A., Griss J., Mayer G., Eisenacher M. (2019). The PRIDE database and related tools and resources in 2019: Improving support for quantification data. Nucleic Acids Res..

[14] Araujo R.L.C., Gönen M., Herman P. (2015). Chemotherapy for Patients with Colorectal Liver Metastases Who Underwent Curative Resection Improves Long-Term Outcomes: Systematic Review and Meta-analysis. Ann. Surg. Oncol..

[15] Ciliberto D., Prati U., Roveda L., Barbieri V., Staropoli N., Abbruzzese A., Caraglia M., Di Maio M., Flotta D., Tassone P. (2012). Role of systemic chemotherapy in the management of resected or resectable colorectal liver metastases: A systematic review and meta-analysis of randomized controlled trials. Oncol. Rep..

[16] Ratti F., Fuks D., Cipriani F., Gayet B., Aldrighetti L. (2019). Timing of Perioperative Chemotherapy Does Not Influence Long-Term Outcome of Patients Undergoing Combined Laparoscopic Colorectal and Liver Resection in Selected Upfront Resectable Synchronous Liver Metastases. World J. Surg..

[17] Said A.H., Raufman J.P., Xie G. (2014). The role of matrix metalloproteinases in colorectal cancer. Cancers.

[18] Koskensalo S., Louhimo J., Nordling S., Hagström J., Haglund C. (2011). MMP-7 as a prognostic marker in colorectal cancer. Tumor Biol..

[19] Vočka M., Langer D., Fryba V., Petrtyl J., Hanus T., Kalousova M., Zima T., Petruzelka L. (2019). Serum levels of TIMP-1 and MMP-7 as potential biomarkers in patients with metastatic colorectal cancer. Int. J. Biol. Markers.

[20] Chen H., Hu Y., Xiang W., Cai Y., Wang Z., Xiao Q., Liu Y., Li Q., Ding K. (2015). Prognostic significance of matrix metalloproteinase 7 immunohistochemical expression in colorectal cancer: A meta-analysis. Int. J. Clin. Exp. Med..

[21] Asano T., Tada M., Cheng S., Takemoto N., Kuramae T., Abe M., Takahashi O., Miyamoto M., Hamada J.I., Moriuchi T. (2008). Prognostic Values of Matrix Metalloproteinase Family Expression in Human Colorectal Carcinoma. J. Surg. Res..

[22] Klupp F., Neumann L., Kahlert C., Diers J., Halama N., Franz C., Schmidt T., Koch M., Weitz J., Schneider M. (2016). Serum MMP7, MMP10 and MMP12 level as negative prognostic markers in colon cancer patients. BMC Cancer.

[23] Macartney-Coxson D.P., Hood K.A., Shi H., Ward T., Wiles A., O’Connor R., Hall D.A., Lea R.A., Royds J.A., Stubbs R.S. (2008). Metastatic susceptibility locus, an 8p hot-spot for tumour progression disrupted in colorectal liver metastases: 13 candidate genes examined at the DNA, mRNA and protein level. BMC Cancer.

[24] Mochizuki S., Okada Y. (2007). ADAMs in cancer cell proliferation and progression. Cancer Sci..

[25] Van Den Eynden G.G., Majeed A.W., Illemann M., Vermeulen P.B., Bird N.C., Høyer-Hansen G., Eefsen R.L., Reynolds A.R., Brodt P. (2013). The multifaceted role of the microenvironment in liver metastasis: Biology and clinical implications. Cancer Res..

[26] Liu Y., Wang G., Liang Z., Mei Z., Wu T., Cui A., Liu C., Cui L. (2018). Lysyl oxidase: A colorectal cancer biomarker of lung and hepatic metastasis. Thorac. Cancer.

[27] Forsburg S.L. (2004). Eukaryotic MCM Proteins: Beyond Replication Initiation. Microbiol. Mol. Biol. Rev..

[28] Ahluwalia P., Mondal A.K., Bloomer C., Fulzele S., Jones K., Ananth S., Gahlay G.K., Heneidi S., Rojiani A.M., Kota V. (2019). Identification and Clinical Validation of a Novel 4 Gene-Signature with Prognostic Utility in Colorectal Cancer. Int. J. Mol. Sci..

[29] Piao C., Cai L., Qiu S., Jia L., Song W., Du J. (2015). Complement 5a enhances hepatic metastases of colon cancer via monocyte chemoattractant protein-1-mediated inflammatory cell infiltration. J. Biol. Chem..

[30] Ding D., Song Y., Yao Y., Zhang S. (2017). Preoperative serum macrophage activated biomarkers soluble mannose receptor (SMR) and soluble haemoglobin scavenger receptor (SCD163), as novel markers for the diagnosis and prognosis of gastric cancer. Oncol. Lett..

