# Supplementary material for: Characterization of Biomarkers in Colorectal Cancer Liver Metastases as a Prognostic Tool
Source: J Pers Med. 2021 Oct 21;11(11):1059. doi: 10.3390/jpm11111059 (PMC8618941; doi:10.3390/jpm11111059)
Supplement: Supplementary file 1 [file jpm-11-01059-s001.zip › jpm-1403087-supplementary.pdf]

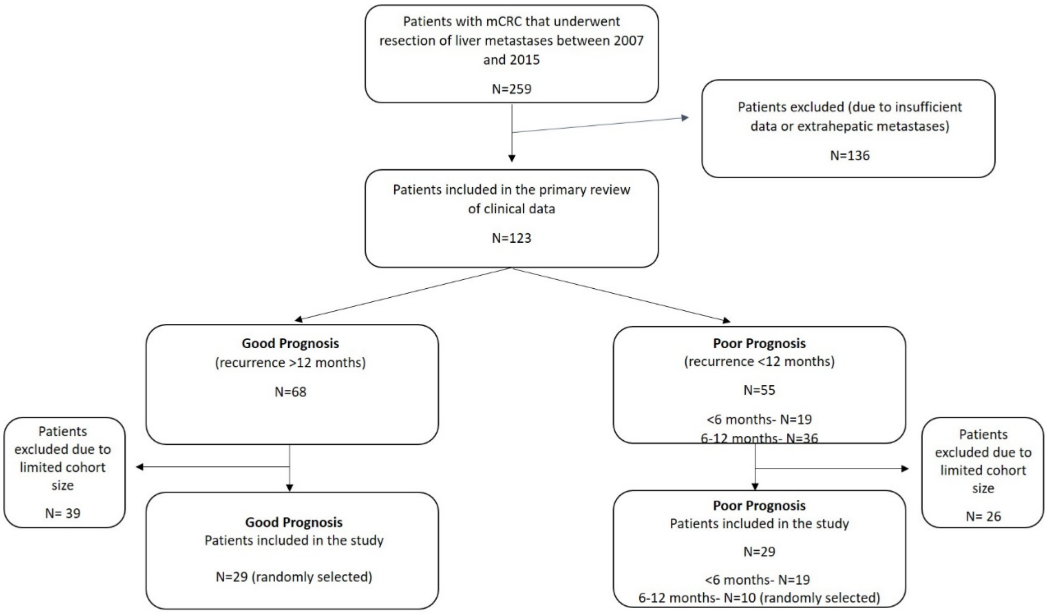

Supplementary Figure S1: Patients inclusion flow-chart.

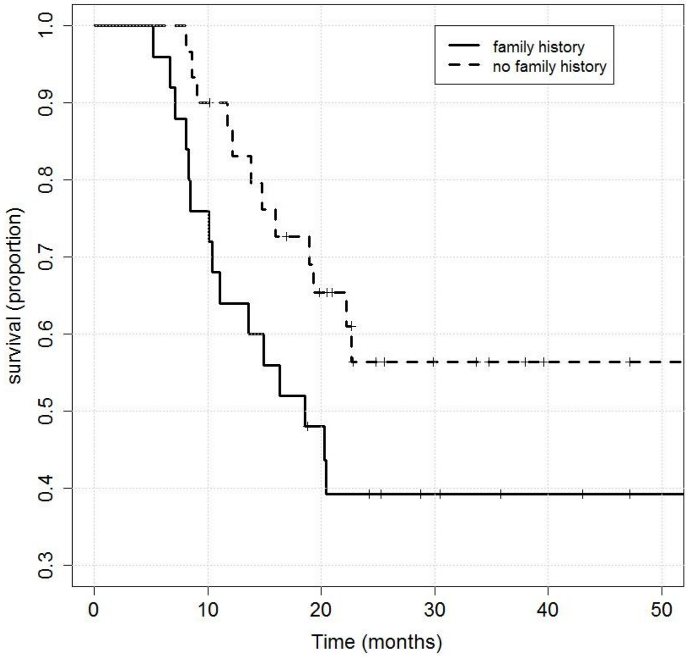

Supplementary Figure S2: KM Survival Curve according to patient's family history – Hazard ratio for disease recurrence within 1 year from surgery in patients without family history was 0.3 [CI: (0.11,0.78), P=0.0144].

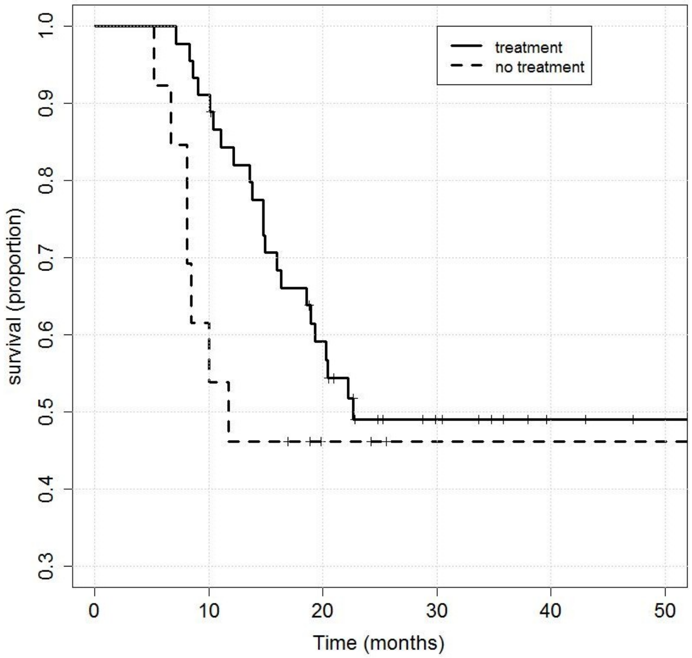

Supplementary Figure S3: KM Survival Curve according to neoadjuvant treatment – The hazard ratio for disease recurrence within 1 year from surgery in patients who did not receive neoadjuvant chemotherapy was 3.83 [CI: (1.12,13.06), P=0.032]. Treatment – Received neoadjuvant treatment. No treatment – Did not receive neoadjuvant treatment.

Supplementary Table S1: List of assessed prognostic factors.

	Good Prognosis	Poor Prognosis	P-value
Gender (M / F)	48% / 52%	62% / 38%	NS
Ethnicity (Ashkenazi Jew, Sephardic Jew, Arab, UNK)	59% / 31% / 7% / 3%	55% / 38% / 3% / 3%	NS
Smoking (Yes / No)	21% / 79%	38% / 62%	NS
Alcohol (Yes / No / UNK)	34% / 66%	10% / 86% / 3%	NS
Diabetes mellitus (Yes / No)	34% / 62% / 3%	28% / 72%	NS
Other Malignancy (Yes / No)	7% / 93%	14% / 86%	NS
Family History of Malignancies (Yes / No / UNK)	35% / 62% / 3%	52% / 41% / 7%	**0.0144**
Primary Tumor Location (Right / Left)	34% / 66%	31% / 69%	NS
LVI (Yes / No / UNK)	34.5% / 34.5% / 32%	17% / 28% / 55%	NS
KRAS (WT / Mut / UNK)	31% / 21% / 48%	21% / 24% / 55%	NS
Synchronous / Metachronous disease	76% / 24%	52% / 48%	NS
Time for metachronous disease appearance (mean month)	32.3	27.2	NS
Median number of livers mets at dg. (Range)	1 (1-4)	1 (1-6)	NS
Median size of larger metastases (Range)	23.5 (10-120) mm	29 (8-160) mm	NS

M – Male / F – Female / UNK – Unknown / LVI - Lymphovascular invasion / WT – Wild type / Mut – mutated / dg. – Diagnosis / NS – Not significant

Supplementary Table S2: List of significantly expressed proteins.

Protein IDs	Protein names	Gene names	ratio (bad vs. good)	FDR adjusted p-value
P10916; G3V1V8; Q9BUA6	Myosin regulatory light chain 2, ventricular/ cardiac muscle isoform	MYL2	1.128218241	0
O43312; H0YB53; E7EWW5	Metastasis suppressor protein 1	MTSS1	1.113171114	0
E9PNM1; A0A1W2PQ47; P37268; E9PJG4; E9PS69	Squalene synthase	FDFT1	1.083580676	3.10139E-11
P13164; H7BYV1; E9PS44; Q01629; Q01628	Interferon-induced transmembrane protein 1; Interferon-induced transmembrane protein 2; Interferon-induced transmembrane protein 3	IFITM1; IFITM2; IFITM3	1.069733709	8.89965E-08
O75610; A0A087WW09	Left-right determination factor 1	LEFTY1	1.068475813	1.70436E-07
Q9BUD6; D6RB12; D6RBY3; D6RIH5	Spondin-2	SPON2	1.065922124	5.88491E-07
P55060	Exportin-2	CSE1L	1.059279627	1.37978E-05
P18065; C9JMY1; C9JW52	Insulin-like growth factor-binding protein 2	IGFBP2	1.055637501	7.53186E-05
E9PHA6; P43246	DNA mismatch repair protein Msh2	MSH2	1.054964008	9.96408E-05
Q96FX7; H0Y2Q1	tRNA (adenine(58)-N(1))-methyltransferase catalytic subunit TRMT61A	TRMT61A	1.053128278	0.000202645
A0A075B6K4; P01715		IGLV3-10	1.052885287	0.00021921
Q6UWY5;	Olfactomedin-like	OLFML1	1.052486124	0.00025455

Protein IDs	Protein names	Gene names	ratio (bad vs. good)	FDR adjusted p-value
E9PN44	protein 1			
P33991; E5RG31	DNA replication licensing factor MCM4	MCM4	1.051558334	0.000343541
Q6PIP6; K7EL65; K7ELF8; O14732	Inositol monophosphatase 2	IMPA2	1.050875085	0.000451896
P10645; G5E968	Chromogranin-A; Vasostatin-1; Vasostatin-2; EA-92; ES-43; Pancreastatin; SS-18; WA-8; WE-14; LF-19; AL-11; GV-19; GR-44; ER-37	CHGA	1.050303356	0.00056478
P07451	Carbonic anhydrase 3	CA3	1.047856586	0.001412062
P15559; B4DLR8; H3BNV2	NAD(P)H dehydrogenase [quinone] 1	NQO1	1.047248185	0.001774045
P53367	Arfaptin-1	ARFIP1	1.045638609	0.003322397
P53814; A0A087WVP4; A0A087X1R1;A0A096LNK9;H7BZZ8;B5MCI0;C9JGQ0;C9JP19; H7C372;C9JQZ8	Smoothelin	SMTN	1.045336474	0.003665062
P09237	Matrilysin	MMP7	1.043704769	0.006367446
A0A0J9YY99; P0DP03; P01768; P01764	Ig heavy chain V-III region CAM; Ig heavy chain V-III region VH26		1.043588544	0.006528347
Q9UBY9; Q68DG0; D3YTC6; Q8N241; C9J5A3	Heat shock protein beta-7	HSPB7; DKFZp779D0968	1.043272005	0.007224123
P16444; H3BQS5; H3BP43	Dipeptidase 1	DPEP1	1.042516677	0.009082335
C9J0J7; G5E9Q6	Profilin	PFN2	1.042114052	0.010057357
P22087; M0R0P1;	rRNA 2-O-methyltransferase	FBL	1.041819482	0.010973843

**Protein IDs**	**Protein names**	**Gene names**	**ratio (bad vs. good)**	**FDR adjusted p-value**
M0R299; M0QXL5; M0R2Q4; M0R2U2; M0R1H0; M0R2B0	fibrillarin			
Q9H0A0; A0A087WV29; E9PMU0; E9PJN6	N-acetyltransferase 10	NAT10	1.041648076	0.011494623
P78539	Sushi repeat-containing protein SRPX	SRPX	1.041432939	0.012234208
Q9NX24; D6RC52; D6RCB9; H0YC83; J3QSY4	H/ACA ribonucleoprotein complex subunit 2	NHP2	1.040990853	0.014135042
H3BRU4; F8W8F7; Q86Y34	Probable G-protein coupled receptor 97	GPR97	1.040362623	0.017163009
Q8NFH5; C9IYQ7; F8WCF5; F8WEL4; C9JIY9; C9JWU7	Nucleoporin NUP53	NUP35	1.040244324	0.017366493
Q9H7D7; H0Y9R3; H0Y917	WD repeat-containing protein 26	WDR26	1.03993936	0.019062689
H3BQV3; Q96MW5	Conserved oligomeric Golgi complex subunit 8	COG8	1.039680004	0.019724425
E7EQ12; D6RAA8; D6RC54		CAST	1.038565883	0.025841849
F5H8H2; Q03426; A0A1B0GWC2; F5GXC0; F5H092; F5H163; F5H368; A0A1W2PS16; A0A1B0GWF3	Mevalonate kinase	MVK	1.038083204	0.02972975
Q8IV56;	Proline-rich protein 15	PRR15	1.038075161	0.02972975

**Protein IDs**	**Protein names**	**Gene names**	**ratio (bad vs. good)**	**FDR adjusted p-value**
C9JQ22				
Q5HYK7	SH3 domain-containing protein 19	SH3D19	1.037921831	0.030596028
Q9UKX2; P13535; P12882; Q9UKX3; E7EX84	Myosin-2; Myosin-8; Myosin-1	MYH2; MYH8; MYH1	1.037659767	0.033025822
O75323; C9K068; C9J7B1; H7C333; F8WBI5	Protein NipSnap homolog 2	GBAS	1.037537186	0.034013629
O00763; F8W8T8; H0YGH5; F5H5C3	Acetyl-CoA carboxylase 2; Biotin carboxylase	ACACB	1.036849768	0.040399464
Q93099; C9JTX9; H7C576; H7C4R8; H7C5G7	Homogentisate 1,2-dioxygenase	HGD	0.969651026	0.049373254
Q9NZP8; H0YFL7; F5H2Z5; F5GWF3	Complement C1r subcomponent-like protein	C1RL	0.969486323	0.047120402
Q8WU76	Sec1 family domain-containing protein 2	SCFD2	0.969459131	0.047120402
P45954	Short/branched chain specific acyl-CoA dehydrogenase, mitochondrial	ACADSB	0.969389093	0.046508708
Q8N5Z0; D6RC56; D6RFY7; D6REB9	Kynurenine/alpha-aminoadipate aminotransferase, mitochondrial	AADAT	0.969207864	0.044127359
P04350; M0QY85; M0R0X0; M0QY37; M0QX14; M0R278; M0QZL7; M0R1I1	Tubulin beta-4A chain	TUBB4A	0.969198619	0.044127359

Protein IDs	Protein names	Gene names	ratio (bad vs. good)	FDR adjusted p-value
Q38LG0; P10635; E7ENE7; A0A0G2JSD7; C1ID54; A0A0G2JLK7; A0A1B0GU72; Q6NWU0; A0A1B0GV74; A0A0U1RRC6; A0A0U1RQD0; A0A0G2JNM9; A0A0G2JLV4; A0A0G2JMD2; A0A087X1C5; H7BY38; A0A0G2JN61; A0A087WXN8; A0A1B0GUD8; A0A1B0GTQ1	Cytochrome P450 2D6	CYP2D6	0.968935725	0.040981207
O75264; K7EKM7	Small integral membrane protein 24	SMIM24	0.968820069	0.040211779
A0A0A0MR14; A0A0A0MRF2; A0A087X1R2; A0A087X0P3; A0A087WV17; A0A0G2JMM7; Q8IYS5	Osteoclast-associated immunoglobulin-like receptor	OSCAR	0.968598077	0.037590056
E9PBP6; P55157; D6REL9	Microsomal triglyceride transfer protein large subunit	MTTP	0.968371342	0.035067483
P04921; A0A0J9YYA0; A0A0J9YWG3; B7Z1Y6; A0A1W2PQF4; Q9UHC6	Glycophorin-C; Contactin-associated protein-like 2	GYPC; CNTNAP2	0.968262166	0.034109233
K7EJ28; Q2QBA2; A0A0A6YY99; O75888; C9JFN2; C9JF68	Tumor necrosis factor ligand superfamily member 13	TNFSF13	0.967751619	0.02972975
P31025; Q5VSP4	Lipocalin-1; Putative lipocalin 1-like protein	LCN1; LCN1P1	0.967268144	0.025841849

Protein IDs	Protein names	Gene names	ratio (bad vs. good)	FDR adjusted p-value
	1			
P37059; H3BS44; H3BRZ6; H3BNN1; H3BQY3	Estradiol 17-beta-dehydrogenase 2	HSD17B2	0.967117173	0.025063431
P23946	Chymase	CMA1	0.96692032	0.02360208
P56192; H0YHL6; H0YI94; H0YI27; F5H2V6; H0YIP0	Methionine--tRNA ligase, cytoplasmic	MARS	0.966896742	0.02360208
A0A0J9YX34; Q9C040	Tripartite motif-containing protein 2	TRIM2	0.966736108	0.022619886
Q07075; H0YA67	Glutamyl aminopeptidase	ENPEP	0.966513801	0.021094035
Q9UF12	Probable proline dehydrogenase 2	PRODH2	0.966508503	0.021094035
P04114; A8MUN2; CON_ENSEMBL:ENSBTAP00000032840	Apolipoprotein B-100; Apolipoprotein B-48	APOB	0.966337166	0.020280491
A0A087X226; Q3KQV9	UDP-N-acetylhexosamine pyrophosphorylase-like protein 1	UAP1L1	0.966190495	0.019724425
P06133; A0A1W2PQJ2; D6RGY0	UDP-glucuronosyltransferase 2B4	UGT2B4	0.966096097	0.019308861
P22760; C9K0E9	Arylacetamide deacetylase	AADAC	0.9660631	0.019308861
Q8IVS8; A0A0C4DGA0; C9JA32; C9J3N5	Glycerate kinase	GLYCTK	0.965656465	0.017320215
P51161	Gastrotropin	FABP6	0.965395383	0.016155447
Q8WXC6	Myeloma-overexpressed gene 2 protein	MYEOV2	0.963981575	0.010057357

**Protein IDs**	**Protein names**	**Gene names**	**ratio (bad vs. good)**	**FDR adjusted p-value**
CON__Q9UE12 ; CON__Q15323; Q15323; Q14532; CON__Q14532; G3V1C2	Keratin, type I cuticular Ha1	KRT31	0.963847476	0.009865999
Q9NR99	Matrix-remodeling-associated protein 5	MXRA5	0.963043841	0.007383715
P55259; I3L4Y3; I3L2Z7; I3L486; I3L3I2	Pancreatic secretory granule membrane major glycoprotein GP2	GP2	0.962950675	0.007238912
Q9BYE9	Cadherin-related family member 2	CDHR2	0.962331848	0.006054494
Q7Z5P4	17-beta-hydroxysteroid dehydrogenase 13	HSD17B13	0.961374324	0.004128494
O15204	ADAM DEC1	ADAMDEC 1	0.961327264	0.004128494
P01031	Complement C5; Complement C5 beta chain; Complement C5 alpha chain; C5a anaphylatoxin; Complement C5 alpha chain	C5	0.957990569	0.001051295
Q9P2I0	Cleavage and polyadenylation specificity factor subunit 2	CPSF2	0.957730146	0.000954475
F5GZZ9; C9JHR8; Q86VB7	Scavenger receptor cysteine-rich type 1 protein M130; Soluble CD163	CD163	0.957418202	0.000845924
Q08397; H3BUV8	Lysyl oxidase homolog 1	LOXL1	0.957389504	0.000845924
O15488; A0A1W2PQ75; J3QSZ3	Glycogenin-2	GYG2	0.955117652	0.000321036
P53582; D6RF24	Methionine aminopeptidase 1	METAP1	0.95510266	0.000321036
P39900	Macrophage metalloelastase	MMP12	0.954669549	0.000272684
Q92626; H7C1W1;	Peroxidasin homolog	PXDN	0.953565247	0.00017794

**Protein IDs**	**Protein names**	**Gene names**	**ratio (bad vs. good)**	**FDR adjusted p-value**
H7C300; C9J4I9				
CON__P78386; P78386; F5GYI5	Keratin, type II cuticular Hb5	KRT85	0.953290248	0.000160117
O60218	Aldo-keto reductase family 1 member B10	AKR1B10	0.952673546	0.000120956
P23378; A0A1W2PP74; A0A1W2PPB1; A0A1W2PQV3; A0A1W2PPE1; A0A1W2PPK8; A0A1W2PQU6; A0A1W2PPH6; A0A1W2PPG0	Glycine dehydrogenase (decarboxylating), mitochondrial	GLDC	0.949066311	1.97473E-05
CON__P02754			0.947845915	1.08456E-05
P07357	Complement component C8 alpha chain	C8A	0.945632919	3.15765E-06
P04745; P19961; P04746; Q5T085; CON__Q3MHH8; H7BZQ8; C9J2Z5; Q5T084; C9JWK7	Alpha-amylase 1; Alpha-amylase 2B; Pancreatic alpha-amylase; Alpha-amylase	AMY1A; AMY2B; AMY2A; AMY1B	0.945212236	2.57897E-06
Q01523	Defensin-5;HD5(23-94);HD5(29-94);HD5(56-94);HD5(63-94)	DEFA5	0.944639327	1.92336E-06
O43490	Prominin-1	PROM1	0.944343592	1.6945E-06
Q04826	HLA class I histocompatibility antigen, B-40 alpha chain	HLA-B	0.943866785	1.33979E-06
O60240; H0YM16	Perilipin-1	PLIN1	0.942433269	5.88491E-07
D6RB89; P50120	Retinol-binding protein 2	RBP2	0.940849049	2.43864E-07
A0A0G2JR65; Q02817; A0A0G2JM87;	Mucin-2	MUC2	0.938034965	4.80026E-08

Protein IDs	Protein names	Gene names	ratio (bad vs. good)	FDR adjusted p-value
Q9UMI9				
P01275	Glucagon; Glicentin; Glicentin-related polypeptide; Oxyntomodulin; Glucagon; Glucagon-like peptide 1; Glucagon-like peptide 1(7-37); Glucagon-like peptide 1(7-36); Glucagon-like peptide 2	GCG	0.934562059	4.74045E-09
P09488; E7EWW9; B9ZVX7; H3BQT3; H3BRM6	Glutathione S-transferase Mu 1	GSTM1	0.931417253	5.27109E-10
Q8NES3; A0A087X191	Beta-1,3-N-acetylglucosaminyltransferase lunatic fringe	LFNG	0.931247289	5.15874E-10
B7ZL91; Q16819	Metalloendopeptidase; Meprin A subunit alpha	MEP1A	0.931140808	5.15874E-10
Q6UX06	Olfactomedin-4	OLFM4	0.927737694	4.47084E-11
A6NCN2	Putative keratin-87 protein	KRT87P	0.919241106	0
O43741	5-AMP-activated protein kinase subunit beta-2	PRKAB2	0.918979831	0
CON__P02663			0.917388047	0
